# E-learning interventions are comparable to user's manual in a randomized trial of training strategies for the AGREE II

**DOI:** 10.1186/1748-5908-6-81

**Published:** 2011-07-26

**Authors:** Melissa C Brouwers, Julie Makarski, Lisa D Durocher, Anthony J Levinson

**Affiliations:** 1 Department of Oncology, McMaster University, Hamilton, Ontario, Canada; 2 Department of Clinical Epidemiology, McMaster University, Hamilton, Ontario, Canada; 3 Division of e-Learning Innovation, McMaster University, Hamilton, Ontario, Canada

## Abstract

**Background:**

Practice guidelines (PGs) are systematically developed statements intended to assist in patient and practitioner decisions. The AGREE II is the revised tool for PG development, reporting, and evaluation, comprised of 23 items, two global rating scores, and a new User's Manual. In this study, we sought to develop, execute, and evaluate the impact of two internet interventions designed to accelerate the capacity of stakeholders to use the AGREE II.

**Methods:**

Participants were randomized to one of three training conditions. 'Tutorial'--participants proceeded through the online tutorial with a virtual coach and reviewed a PDF copy of the AGREE II. 'Tutorial + Practice Exercise'--in addition to the Tutorial, participants also appraised a 'practice' PG. For the practice PG appraisal, participants received feedback on how their scores compared to expert norms and formative feedback if scores fell outside the predefined range. *'*AGREE II User's Manual PDF (control condition)'*--*participants reviewed a PDF copy of the AGREE II only. All participants evaluated a test PG using the AGREE II. Outcomes of interest were learners' performance, satisfaction, self-efficacy, mental effort, time-on-task, and perceptions of AGREE II.

**Results:**

No differences emerged between training conditions on any of the outcome measures.

**Conclusions:**

We believe these results can be explained by better than anticipated performance of the AGREE II PDF materials (control condition) or the participants' level of health methodology and PG experience rather than the failure of the online training interventions. Some data suggest the online tools may be useful for trainees new to this field; however, this requires further study.

## Background

Evidence-based practice guidelines (PGs) are systematically developed statements aimed at assisting clinicians and patients to make decisions about appropriate healthcare for specific clinical circumstances [1] and to inform decisions made by policy makers [2-4]. While PGs have been shown to have a moderate impact on behavior [5], their potential for benefit is only as good as the PGs themselves [6-8]. The AGREE II, a revised version of the original tool [9], is an instrument designed to direct the development, reporting, and evaluation of PGs [10-13]. The AGREE II consists of 23 items grouped into six quality domains, two overall assessment items, and extensive supporting documentation to facilitate its appropriate application (*i.e*., User's Manual).

International adoption of the original AGREE Instrument and interest in the revised version has been significant, and attests to the potential value of this tool [14]. The AGREE II was designed for many different types of users and for users with varied expertise. Given the breadth and heterogeneity of the AGREE II's stakeholder group, efforts to promote and facilitate its application are complex. The internet is a key medium to reach a vast, varied, and global audience. However, passive internet dissemination alone, even with a primed and interested audience, will not fully optimize its application and use. Our interest was to explore educational interventions and to leverage technical platforms to accelerate an effective application process.

E-learning (internet-based training) provides a potentially effective, standardized, and cost-efficient model for training in the use of AGREE II. A recent meta-analysis and systematic review showed large effect sizes for internet-based instruction (clinical and methodological content areas) on outcomes with health-profession learners [15,16]. Improved learning outcomes seemed to be associated with designs that included interactivity, practice exercises, repetition, and feedback. Thus, e-learning appeared to be a promising solution for our context. While the evidence base underpinning the efficacy and design principles of e-learning training materials are well established [17-23], there remain questions regarding the optimal application and combination of these principles for particular interventions. In this study, we wanted to design and test two e-learning interventions, a tutorial alone versus a tutorial plus an interactive practice exercise, against a more traditional learning form to determine their impact on outcomes related to the AGREE II.

Our primary research question is, whether compared to just reading the User's Manual, does the addition of an online tutorial program, with or without a practice exercise with feedback, improve learners' performance and increase learners' satisfaction and self-efficacy with the AGREE II? Based on the results of systematic reviews [15,16], we hypothesized the training platform that included the tutorial plus the practice exercise with feedback would be superior to the User's Manual alone. For exploratory purposes, we also examined whether differences existed across the outcome measures between the two e-learning intervention groups.

## Methods

This study was funded by the Canadian Institutes of Health Research and received ethics approval from the Hamilton Health Sciences/Faculty of Health Sciences Research Ethics Board (REB #09-398; McMaster University, Hamilton, Ontario, Canada). Key evidence-based principles in the science of technical training, multimedia learning, and cognitive psychology were used to develop the two training platforms [17-23].

### Study design and intervention

**Figure 1:**
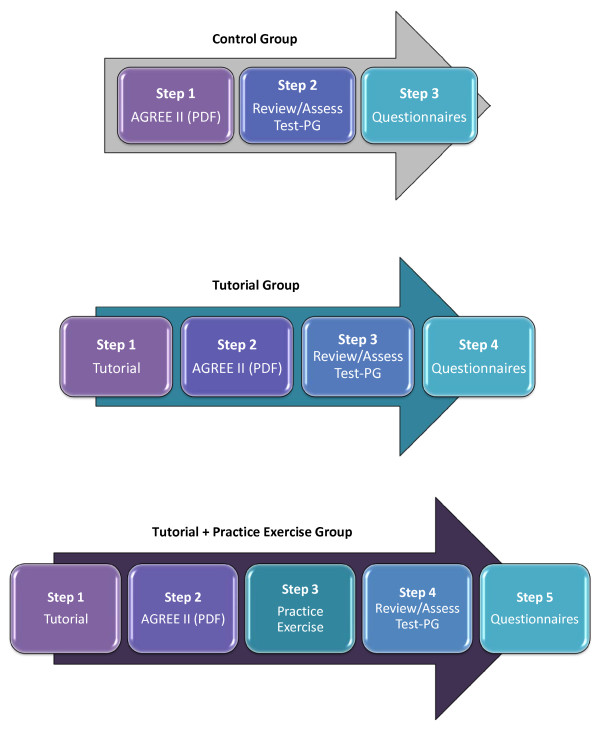
**Study description**.

A single factorial design with three levels of training intervention was implemented (see Figure 1).

### Tutorial

Participants received access to a password-protected website where they were presented with a seven-minute multimedia tutorial presentation with an overview of the AGREE II conducted by a 'virtual coach.' Following the tutorial, the participants were granted access to a PDF copy of the AGREE II and were instructed to review the User's Manual before proceeding to the test PG.

### Tutorial + practice exercise

Participants received access to a password-protected website where they received the same tutorial presentation described above and access to the AGREE II User's Manual. They were then presented with the practice exercise that required participants to read a sample or 'practice' PG and appraise it using the AGREE II. Upon entering each AGREE II score, participants were provided immediate feedback on how their score compared to the mean of four experts. If their score fell outside a predefined range, participants received two-stage formative feedback to guide the appraisal process. At the conclusion of their review, participants received a summary of their performance in appraising the practice PG compared to expert norms. Participants then proceeded to read and appraise the test PG.

### User's manual

Participants assigned to the control condition received PDF copies of the AGREE II User's Manual for review before proceeding to the test PG. The User's Manual is a 56-page document. It provides an overview of the AGREE enterprise and general instructions on how to use the tool. Then, for each of the 23 core items, it presents a definition of the concept and examples, advice on where the information can be found within a PG document, and the specific criteria and considerations for scoring. It concludes with the two global rating measures.

### Participants and process

Following our sample size calculation reported in the detailed protocol previously published [14], we required 20 participants per group to have at least 80% power to detect a performance advantage of as little as ± 0.79 standard deviations for either of the intervention groups compared to the passive learning group. Methodologists, clinicians, policy makers, and trainees were sought from guideline programs, professional directories, and the Guidelines International Network (G-I-N) community. Because our previous research showed virtually no differences in AGREE II performance as a function of type of users, we did not account for this factor in our study design [11-13].

A total of 107 interested individuals registered with the Scientific Office. After receiving a letter of invitation and screening for their eligibility, 87 participants were randomized to one of the three training conditions using a computer-generated randomization sequence (1:1:1 ratio). Individuals were eligible for study participation if they had no or limited experience and exposure to the original AGREE Instrument or the AGREE II. To assess this, participants were asked to first complete an online eligibility questionnaire. Here, they were asked about the type(s) of previous experience they had with the original AGREE and AGREE II (as a tool to inform guideline development, guideline reporting, guideline evaluation, and other) and the extent of this experience (never, 1 to 5 guidelines, 6 to 10 guidelines, 11 to 15 guidelines, 16 to 20 guidelines, 20+ guidelines). They were also asked if they had participated in any AGREE-related research study previously (yes, no, uncertain). Participants who answered they had not participated in an AGREE-related research study and who had little to no AGREE or AGREE II experience (defined as never using either instrument or using it on a maximum of 1 to 5 guidelines) were eligible to participate.

These individuals were then randomized to group and received access to an individualized password-protected web-based study platform. Participants completed their specific training intervention, evaluated one of ten test PGs using the AGREE II, and completed a series of post-test Learner's Scales and a demographics survey. Participants were blinded to the study conditions, our research questions, and hypothesis.

## Materials and instruments

### Practice guidelines

Eleven PGs were selected for this study: one served as the practice PG for participants randomized to the Tutorial + Practice Exercise group and, to facilitate generalizability of results, the remaining ten were selected for the test PGs. Participants were randomized to one of the ten test PGs. Practice guideline was not a factor of analytic interest. Eligibility criteria for the 11 PGs are described in detail in the previously published protocol and include: English-language documents published from 2002 onward; were within the clinical areas of cancer, cardiovascular, or critical care; were 50 pages or less; and represented a range of quality [14].

### AGREE II performance

The AGREE II consists of survey items and a User's Manual [11-13]: twenty-three items are grouped into six domains of PG quality: scope and purpose, stakeholder involvement, rigour of development, clarity of presentation, applicability, and editorial independence. Items are answered using a 7-point response scale ('strongly disagree' to 'strongly agree'). Standardized domain scores are calculated enabling construction of a performance score profile permitting direct comparisons across the domains or items. The AGREE II survey items conclude with two global measures answered using a 7-point scale: one item targeting the PG's overall quality and the second targeting the appraiser's intention to use the PG. The User's Manual provides explicit direction for each of the 23 and two overall items, as noted above. Participant performance served as the primary outcome.

### Learner's scale

In addition to the primary outcome of performance on the test PG, a series of secondary measures, known as the Learner's scale, were also collected. This scale was comprised of Learner Satisfaction scale (*i.e*., satisfaction with learning opportunity), Self-Efficacy scale (*i.e*., belief one can succeed), Mental Effort scale (*i.e*., cognitive effort to complete a task), and Time-on-Task. With the exception of Time-on-Task, which was a self-report measure, a 7-point response scale was used to answer the remaining items. The questions included in the Learner's scale were inspired by previous work done in this field [17-23]. Specific reliability and validity testing of the items and subscales was not undertaken.

### AGREE II perceptions

Participants were asked to rate the usefulness of the AGREE II (for development, reporting, and evaluation) and the User's Manual using a 7-point scale.

### Demographics and AGREE II Experience scale

Participants were asked about their backgrounds including experience with the PG enterprise, the original AGREE instrument and the AGREE II.

### Outcomes and analyses

#### Primary measures

Two performance measures served as the primary outcomes. First, the Performance - Distance Function calculates the difference between the domain scores of the participants from those of expert norms. Expert norms were derived by members of the AGREE Next Steps research team who appraised the test PGs used in this study. Four expert appraisers rated each guideline. Mean standardized scores were used to construct the expert performance score profiles. Thus, the measure of distance (*i.e*., difference in scores between participants and experts) for each AGREE II domain was calculated by squaring the difference between the participants' profile domain ratings from the experts' profile domain ratings. A series of one-way analysis of variance tests were subsequently calculated to examine differences in distance function as a function of training intervention.

Second, performance was measured by examining the proportion of participants who met minimum performance competencies with the AGREE II tool [14]. A Pass/Fail algorithm designed for another study [14] was used here to calculate the performance level for participants randomized to the condition with the practice PG.

#### Secondary measures

The Learner's scale served as the core secondary measure. To this end, a series of multivariate one-way analysis of variance tests were conducted to examine differences in participants' satisfaction, self-efficacy, and mental effort as a function of training intervention. A series of analysis of variance tests were conducted to examine differences in participants' self-reported Time-on-Task and in participants' reported perceptions of the AGREE II.

## Results

There were no changes to any of the outcomes once the trial commenced.

### Participants (Table 1 and Figure 2)

**Table 1:** Demographics

	Group 1: Tutorial	Group 2: Practice Exercise	Group 3: Control
Gender			

% Female	75%	60%	55%

Age			

18 to 24	0%	20%	0%

25 to 34	35%	15%	15%

35 to 44	15%	30%	35%

45 to 54	30%	30%	30%

55 to 64	20%	15%	20%

Participants' Training			

Education			

Bachelors	95%	65%	80%

Masters	45%	50%	45%

PhD	25%	15%	5%

Physician	30%	35%	30%

Registered Nurse	15%	20%	15%

Allied Health (*e.g.*, PT, OT, RT)	5%	10%	0%

Other (non specified)	0%	10%	5%

% with health research methods training			

	85%	85%	100%

Previous Experience			

Use of AGREE as a tool to inform PG development			

Never	78%	65%	65%

1 to 5 times	10%	25%	22%

Use of AGREE as a tool to inform PG reporting			

Never	75%	74%	76%

1 to 5 times	15%	10%	17%

Use of AGREE as a tool to evaluate PG			

Never	71%	61%	48%

1 to 5 times	16%	26%	35%

Use of AGREE II as a tool to inform PG development			

Never	94%	94%	84%

1 to 5 times	6%	6%	10%

Use of AGREE II as a tool to inform PG reporting			

Never	97%	100%	84%

1 to 5 times	3%	0%	10%

Use of AGREE II as a tool to evaluate PG			

Never	94%	94%	84%

1 to 5 times	6%	6%	10%

**Figure 2:**
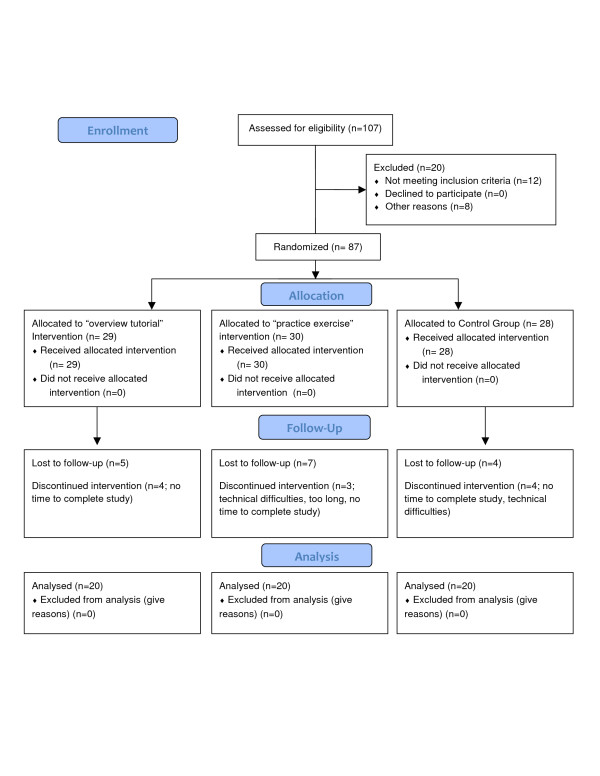
**CONSORT flow diagram**.

Letters of invitation were sent to 107 participants, of which 87 were eligible to participate (12 were excluded based on past experience with the AGREE Instrument and eight were non-respondents to the letter of invitation). Sixty participants completed the study (response rate = 69%), 20 per condition. The majority of participants were female, between the ages of 25 and 65, and with some level of health methods training.

### Performance - distance function (Table 2)

**Table 2:** Distance function (mean (standard deviation))*

Domain	Overview Tutorial	Practice Exercise	Control	Sig
Domain 1. Scope and Purpose	3.21 (2.96)	2.61 (2.96)	1.90 (2.64)	0.36

Domain 2. Stakeholder Involvement	1.68(2.63)	2.03 (2.36)	1.71 (2.54)	0.89

Domain 3. Rigour of Development	1.90 (3.26)	1.85 (3.20)	1.02 (1.40)	0.53

Domain 4. Clarity of Presentation	0.93 (1.11)	2.86 (4.43)	2.14 (2.15)	0.12

Domain 5. Applicability	3.03 (3.77)	1.92 (2.83)	2.05 (2.28)	0.45

Domain 6. Editorial Independence	3.17 (4.41)	2.84 (4.66)	1.86 (3.50)	0.60

*Distance Function = (mean domain score experts - mean domain score of participants)^2 ^Larger numbers denote greater difference between participant and expert performance.

There were no significant differences in any of the domain distance functions between the three training groups (*p *> 0.05 for all comparisons).

### Performance - pass/fail criteria

86% of the individuals in the Tutorial + Practice Exercise training intervention arm passed the online training with the practice PG.

### Training satisfaction and self-efficacy (Table 3)

**Table 3:** Training Satisfaction and Self-Efficacy Ratings (1 to 7 scale; means and (standard deviations)).

Training Satisfaction and Self-Efficacy	Overview Tutorial	Practice Exercise	Control	Univariate Sig
Training Satisfaction (MANOVA, *p *> 0.05)				

The training exercise was conveyed at the appropriate level	5.85 (1.09)	6.16 (0.77)	5.95 (0.83)	0.67

The training exercise was a valuable learning experience	6.10 (0.78)	6.35 (0.75)	5.75 (1.12)	0.15

The training exercise was a positive experience	6.00 (0.80)	6.05 (0.95)	5.45 (1.32)	0.09

The training exercise was completed in a reasonable amount of time	5.10 (1.74)	4.60 (1.96)	5.65 (1.23)	0.16

The training exercise has increased my understanding of the content of the AGREE II	6.45 (0.83)	6.40 (1.00)	6.25 (0.79)	0.77

The training exercise has increased my confidence to assess the quality of PGs using the AGREE II	5.85 (0.99)	5.95 (0.95)	6.00 (0.80)	0.87

I was able to navigate the training exercise with ease	6.30 (1.03)	6.11 (0.94)	6.15 (1.09)	0.89

The information in the training exercise was logically grouped together	6.35 (0.81)	6.45 (0.76)	6.30 (0.80)	0.85

The training exercise achieved its stated objectives.	6.25 (0.72)	5.95 (0.95)	5.80 (0.95)	0.26

The training exercise was relevant to my practice/goals and my learning needs.	6.10 (0.85)	6.30 (0.98)	5.70 (1.13)	0.15

Overall, I was satisfied with my AGREE II training experience	6.15 (0.75)	6.05 (1.15)	6.1 (0.72)	0.98

*Self-Efficacy (MANOVA, p *> 0.05)				

I am confident in my ability to use the AGREE II to assess PGs	5.20 (1.28)	5.25 (0.91)	5.60 (1.10)	0.47

I am comfortable with the structure of the AGREE II	5.80 (0.77)	6.10 (0.72)	6.05 (0.69)	0.38

I am comfortable with the content of the AGREE II	5.80 (0.83)	5.65 (0.59)	6.00 (0.73)	0.31

I am confident in applying my AGREE II skills	5.20 (1.20)	5.20 (1.05)	5.65 (0.99)	0.29

Participants reported high levels of training satisfaction (means 6.0+) and self-efficacy (means 5.4+). There were no significant differences in any measure as a function of training condition (*p *> 0.05 for all comparisons). The Tutorial, Tutorial + Practice Exercise, and review of the PDF training options were recommended by 80%, 60%, and 60% of participants, respectively (*p *> 0.05 for all comparisons).

### Mental effort (Table 4)

**Table 4:** Mental Effort Ratings (1 to 7 scale; means (standard deviations)).

Mental Effort (MANOVA, *p *> 0.05)	Tutorial	Practice Exercise	Control	Univariate Sig
Mental effort tutorial: The AGREE II Overview Tutorial was mentally demanding	3.65 (1.50)	2.85 (1.81)	-	0.14

Mental effort tutorial: The pace of the AGREE II Overview Tutorial was hurried/rushed	2.95 (1.57)	2.60 (1.61)	-	0.54

At the end of the AGREE II Overview Tutorial, I was discouraged	2.30 (1.46)	2.15 (1.53)	-	0.75

Reviewing the AGREE II was mentally demanding	4.40 (1.50)	3.10 (1.25)	4.37 (1.46)	0.006

At the end of reviewing the AGREE II, I was discouraged	3.20 (1.77)	2.30 (1.53)	2.25 (1.25)	0.10

The interactive practice exercise was mentally demanding	-	4.55 (1.40)	-	-

At the end of the interactive practice exercise, I was discouraged	-	3.16 (1.80)	-	-

Rating and assessing the practice guideline with the AGREE II was mentally demanding	4.85 (1.50)	4.70 (1.46)	5.05 (1.54)	0.76

Rating and assessing the practice guideline with the AGREE II was very hard work	4.25 (1.69)	3.90 (1.56)	3.50 (1.91)	0.39

At the end of rating and assessing the practice guideline with the AGREE II, I was discouraged	2.95 (1.57)	2.65 (1.63)	2.30 (1.17)	0.38

The multivariate analysis of variance failed to show a difference in participants' reporting of mental effort as a function of training condition. With the exception of one measure (the AGREE II was mentally demanding), the univariate analyses of variance also failed to show significance differences.

### Time-on-task (Table 5)

**Table 5:** Time-on-Task (minutes; means (standard deviations)).

Time-on-Task	Overview Tutorial	Practice Exercise	Control	Sig
User's rating of how long it took to overview PDF copy of AGREE II	31.70 (24.97)	29.1 (22.83)	38.4 (18.30)	0.40

User's rating of how long it took to do interactive practice exercise	-	51.9 (30.05)	-	-

User's rating of how long it took to read and rate PG	70.50 (52.91)	61.9 (29.48)	75.55 (50.02)	0.63

There were no significant differences as a function of training condition in the time spent by participants reviewing either the PDF version of the AGREE II or in the time taken to complete the test PG (*p *> 0.05 for all comparisons).

### AGREE II perceptions (Table 6)

**Table 6:** AGREE II Perceptions (1 to 7 scale; means and (standard deviations)).

AGREE II Perception	Overview Tutorial	Practice Exercise	Control	Sig
I believe the AGREE II will be a useful tool to inform practice guideline development	6.45 (0.605)	6.20 (0.70)	6.25 (0.72)	0.47

I believe the AGREE II will be a useful tool to inform practice guideline reporting	6.40 (0.60)	6.25 (0.79)	6.05 (0.76)	0.31

I believe the AGREE II will be a useful tool to evaluate practice guidelines	6.50 (0.61)	6.50 (0.61)	6.25 (0.72)	0.37

I believe the User's Manual enhanced my skill in use of applying the AGREE II	5.90 (0.80)	5.95 (1.05)	6.00 (0.92)	0.94

Participants reported favourable perceptions about the AGREE II as a tool to facilitate the development, reporting, and evaluation of PGs; they also reported favourable perceptions about the AGREE II User's Manual in enhancing skills with its application. No significant differences were found for any outcome as a function of training intervention conditioSn.

## Discussion

In this study, we tested two internet-based electronic training interventions against a traditional training method using a PDF version of the User's Manual to determine their effects on various measures related to performance on and attitudes toward the AGREE II. The goal was to identify the best strategy to facilitate the AGREE II's appropriate and effective uptake by its stakeholders. In contrast to our hypotheses, participants randomized to the training condition that included the Tutorial + Practice Exercise did not demonstrate superior performance with the AGREE II, greater satisfaction with the training experience, higher levels of self-efficacy, or more positive attitudes toward the tool than did participants randomized to the other two conditions.

One potential explanation is that our randomization did not work properly, and there were differences in experience participants had in health research methodology and/or the AGREE or the AGREE II. Our demographic data (see Table 2) suggest participants allocated to the control condition may have been more apt to have had minimal exposure than no exposure to the tools than were participants allocated to the other conditions. The inclusion of direct pretest measures to more accurately capture guideline performance before training exposure and to ensure baseline characteristics of the participants do not vary on this factor may be warranted in future studies.

A second potential explanation for our findings is that our interventions did not work. This explanation, however, is not well supported. First, each intervention arm aligned with design characteristics found in other studies and systematic reviews to be effective training features, such as immediate feedback, interactivity, and repetition [15,16]. Second, albeit the data are subjective, they do show that participants liked all of our interventions; for example, satisfaction measures and self-efficacy measures are extremely high, well above the mid-point of the 7-point response scale. To that end, one may conclude then, that our control condition (*i.e*., review of the PDF version of the AGREE II only) was very effective, and that there is a ceiling effect on performance measures and other outcomes.

Exploring these conclusions further, a significant component in the revision of the AGREE II was the reworking of the User's Manual and its written training resource component. As described, the document provides descriptions, examples, and explicit direction for how to evaluate a PG report using AGREE II. The comprehensive nature of the PDF version of the AGREE II User's Manual may be quite sufficient for many potential users. In fact, previous research, as was found in this study, demonstrates high support for the User's Manual by participants [13].

While this study failed to demonstrate superiority of the online electronic training interventions, we do not believe they should be abandoned all together. While we were successful in screening participants so that they had little-to-no experience with the AGREE II or the original version of the tool, virtually all participants had some experience in health methods (*e.g. *systematic review, critical appraisal) and many had experience with the PG enterprise (see Table 1). This selection bias may represent a limitation to the study that also compromises the interpretability of the findings. Specifically, it may be that the online training interventions would be of benefit to the truly novice participant: individuals with no experience with the AGREE II, PGs in general, or health research methodology--for example, trainees and students in the field of health services research. There are some previous data to support this. In the separate project that developed the pass-fail algorithm used in this study, most of the participants were trainees early on in their post-graduate career with considerably less experience in health methods or PGs. In contrast to pass rates of 86% reported in this study, the initial pass rates for those participants was 73%, suggesting the training may be better suited for novice users. Future research studies recruiting these types of participants are warranted.

Indeed, educational research supports the notion of adapting instructional methods based on individual differences in prior knowledge. In general, the literature suggests that good instructional design techniques may be of more importance for low prior knowledge than for high prior knowledge learners [19,22]. Redundant content should usually be eliminated for more experienced learners. It is possible that the more knowledgeable learners in our study experienced unnecessary extra cognitive load from the additional e-learning instructional interventions, when the control materials of the User's Manual were sufficient. There may even be expertise reversal effects, where a given instructional method that works well for novice learners [24] is less effective or even detrimental for individuals with more expertise [25]. In this study, it is possible that either the ceiling effect or detrimental effects of redundancy may have led to no difference from the control condition. Further investigation is required to assess whether efficient instruction on the AGREE II for more advanced learners will require different methods than training designed for entry-level learners.

In summary, our study did not demonstrate our two online AGREE II electronic training interventions improved outcomes over the control condition. We believe this can be explained in part by the better than expected performance of the control condition (*i.e*. current standard of the PDF AGREE II, namely the User's Manual) and in part by the level of experience among the participants with health methods and PGs. Future research may demonstrate that the two online training interventions may be best suited to and effective tools for very novice users, new to the area of PGs and the AGREE II Enterprise. The training interventions are available through the AGREE Enterprise Web site [26].

## Competing interests

The authors declare that they have no competing interests.

## Authors' contributions

MCB conceived of the concept and design of the originally funded proposal, oversaw the project execution and data analyses, drafted and revised this manuscript, and has given final approval for the manuscript to be published. JM contributed to the design of the originally funded proposal, oversaw the project execution and data analyses, contributed substantially to the revisions of the manuscript, and has given final approval for the manuscript to be published. LDD contributed to data collection, data analyses, contributed substantially to the revisions of the manuscript, and has given final approval for the manuscript to be published. AJL contributed to the design of the originally funded proposal, contributed substantially to the revisions of the manuscript, and has given final approval for the manuscript to be published. He led the instructional design and building of the overview tutorial interventions.

## References

[B1] Committee to Advise the Public Health Service on Clinical Practice Guidelines, Institute of Medicine Field MJ Lohr KN (Eds) Clinical practice guidelines: directions for a new program 1990 Washington: National Academy Press

[B2] Browman GP Snider A Ellis P The healthcare manager as catalyst for evidence-based practice: changing the healthcare environment and sharing experience Healthc Pap 2003 3 3 10 22 12811083 10.12927/hcpap..17125

[B3] Browman GP Snider A Ellis P Transferring knowledge and effecting change in working healthcare environments: Response to seven commentaries Healthc Pap 2003 3 3 66 71

[B4] Browman GP Brouwers M Fervers B Sawka C Elwood JM, Sutcliffe SB Population-based cancer control and the role of guidelines-towards a 'systems' approach Cancer Control 2010 Oxford: Oxford University Press

[B5] Francke AL Smit MC de Veer AJE Mistiaen P Factors influencing the implementation of clinical guidelines for health care professionals: A systematic meta-review BMC Med Inform Dec Mak 2008 8 38 10.1186/1472-6947-8-38 PMC2551591 18789150

[B6] Grimshaw JM Thomas RE MacLennan G Fraser C Ramsay CR Vale L Whitty P Eccles MP Matowe L Shirran L Wensing M Dijkstra R Donaldson C Effectiveness and efficiency of guideline dissemination and implementation strategies Health Technol Assess 2004 8 6 iii iv 1-72. [Review] 14960256 10.3310/hta8060

[B7] Cabana M Rand CS Powe NR Wu AW Wilson MH Abboud PAC Rubin HR Why don't physicians follow clinical practice guidelines? JAMA 1999 282 1458 1465 10.1001/jama.282.15.1458 10535437

[B8] Schünemann HJ Fretheim A Oxman AD Improving the use of research evidence in guideline development: 13. Applicability, transferability and adaptation Health Res Policy Syst 2006 4 25 10.1186/1478-4505-4-25 17156457 PMC1712227

[B9] Streiner DL Norman GR Health Measurement Scales. A practical guide to their development and use 2003 3 Oxford: Oxford University Press

[B10] Cluzeau F Burgers J Brouwers M Grol R Makela M Littlejohns P Grimshaw J Hunt C for the AGREE Collaboration Development and validation of an international appraisal instrument for assessing the quality of clinical practice guidelines: the AGREE project Qual Safe Health Care 2003 12 18 23 10.1136/qhc.12.1.18 PMC1743672 12571340

[B11] Brouwers M Kho ME Browman GP Burgers JS Cluzeau F Feder G Fervers B Graham ID Grimshaw J Hanna S Littlejohns P Makarski J Zitzelsberger L for the AGREE Next Steps Consortium AGREE II: Advancing guideline development, reporting and evaluation in healthcare Can Med Assoc J 2010 182 E839 E842 10.1503/cmaj.090449 PMC3001530 20603348

[B12] Brouwers MC Kho ME Browman GP Burgers J Cluzeau F Feder G Fervers B Graham ID Hanna SE Makarski J on behalf of the AGREE Next Steps Consortium Performance, Usefulness and Areas for Improvement: Development Steps Towards the AGREE II - Part 1 Can Med Assoc J 2010 182 1045 1052 10.1503/cmaj.091714 PMC2900328 20513780

[B13] Brouwers MC Kho ME Browman GP Burgers J Cluzeau F Feder G Fervers B Graham ID Hanna SE Makarski J on behalf of the AGREE Next Steps Consortium Validity Assessment of Items and Tools To Support Application: Development Steps Towards the AGREE II - Part 2 Can Med Assoc J 2010 182 E472 E478 10.1503/cmaj.091716 PMC2900368 20513779

[B14] Brouwers MC Makarski J Levinson A A randomized trial to evaluate e-learning interventions designed to improve learner's performance, satisfaction, and self-efficacy with the AGREE II Implement Sci 2010 5 29 10.1186/1748-5908-5-29 20403188 PMC2868048

[B15] Cook DA Levinson AJ Garside S Dupras DM Erwin PJ Montori VM Internet-based learning in the health professions: a meta-analysis JAMA 2008 300 1181 1196 10.1001/jama.300.10.1181 18780847

[B16] Cook DA Levinson AJ Garside S Dupras DM Erwin PJ Montori VM Instructional design variations in internet-based learning for health professions education: a systematic review and meta-analysis Acad Med 2010 85 909 922 10.1097/ACM.0b013e3181d6c319 20520049

[B17] Dick W Carey L Carey JO The Systematic Design of Instruction 2005 Boston; Pearson

[B18] Clark RC Developing Technical Training 2008 San Francisco: John Wiley & Sons

[B19] Clark RC Nguyen F Sweller J Efficiency in Learning 2006 San Francisco: John Wiley & Sons

[B20] Clark RC Mayer RE E-Learning and the Science of Instruction 2007 San Francisco: Pfeiffer

[B21] Clark RC Building Expertise 2003 Silver Spring: International Society for Performance Improvement

[B22] Mayer RE Multimedia Learning 2001 New York: Cambridge University Press

[B23] van Merrienboer JJG Sweller J Cognitive load theory in health professional education: design principles and strategies Medical Education 2010 44 85 93 10.1111/j.1365-2923.2009.03498.x 20078759

[B24] Kalyuga S Ayres P Chandler P Sweller J The expertise reversal effect Educ Psychol 2003 38 23 31 10.1207/S15326985EP3801_4

[B25] Kalyuga S Chandler P Tuovinen J Sweller J When problem solving is superior to studying worked examples J Educ Psychol 2001 93 579 588

[B26] AGREE Enterprise Website http://www.agreetrust.org/resource-centre/training/

